# Denitrification potential of the eastern oyster microbiome using a 16S rRNA gene based metabolic inference approach

**DOI:** 10.1371/journal.pone.0185071

**Published:** 2017-09-21

**Authors:** Ann Arfken, Bongkeun Song, Jeff S. Bowman, Michael Piehler

**Affiliations:** 1 Department of Biological Sciences, Virginia Institute of Marine Science, Gloucester Point, Virginia, United States of America; 2 Integrative Oceanography Division, Scripps Institution of Oceanography, University of California, San Diego, California, United States of America; 3 Institute of Marine Sciences, University of North Carolina at Chapel Hill, Morehead City, North Carolina, United States of America; Chengdu Institute of Biology, CHINA

## Abstract

The eastern oyster (*Crassostrea virginica*) is a foundation species providing significant ecosystem services. However, the roles of oyster microbiomes have not been integrated into any of the services, particularly nitrogen removal through denitrification. We investigated the composition and denitrification potential of oyster microbiomes with an approach that combined 16S rRNA gene analysis, metabolic inference, qPCR of the nitrous oxide reductase gene (*nosZ*), and N_2_ flux measurements. Microbiomes of the oyster digestive gland, the oyster shell, and sediments adjacent to the oyster reef were examined based on next generation sequencing (NGS) of 16S rRNA gene amplicons. Denitrification potentials of the microbiomes were determined by metabolic inferences using a customized denitrification gene and genome database with the paprica (PAthway PRediction by phylogenetIC plAcement) bioinformatics pipeline. Denitrification genes examined included nitrite reductase (*nir*S and *nir*K) and nitrous oxide reductase (*nos*Z), which was further subdivided by genotype into clade I (*nos*ZI) or clade II (*nos*ZII). Continuous flow through experiments measuring N_2_ fluxes were conducted with the oysters, shells, and sediments to compare denitrification activities. Paprica properly classified the composition of microbiomes, showing similar classification results from Silva, Greengenes and RDP databases. Microbiomes of the oyster digestive glands and shells were quite different from each other and from the sediments. The relative abundance of denitrifying bacteria inferred by paprica was higher in oysters and shells than in sediments suggesting that oysters act as hotspots for denitrification in the marine environment. Similarly, the inferred *nos*ZI gene abundances were also higher in the oyster and shell microbiomes than in the sediment microbiome. Gene abundances for *nos*ZI were verified with qPCR of *nosZ*I genes, which showed a significant positive correlation (*F*_*1*,7_ = 14.7, p = 6.0x10^-3^, *R*^2^ = 0.68). N_2_ flux rates were significantly higher in the oyster (364.4 ± 23.5 μmol N-N_2_ m^-2^ h^-1^) and oyster shell (355.3 ± 6.4 μmol N-N_2_ m^-2^ h^-1^) compared to the sediment (270.5 ± 20.1 μmol N-N_2_ m^-2^ h^-1^). Thus, bacteria carrying *nos*ZI genes were found to be an important denitrifier, facilitating nitrogen removal in oyster reefs. In addition, this is the first study to validate the use of 16S gene based metabolic inference as a method for determining microbiome function, such as denitrification, by comparing inference results with qPCR gene quantification and rate measurements.

## Introduction

Chesapeake Bay, the largest estuary in the United States, is one of many systems that has experienced the detrimental effects of excess nitrogen (N) and cultural eutrophication, including bottom water hypoxia, reduced fisheries harvests, and loss of submerged aquatic vegetation [1,2]. Over the last several years, restoration of the eastern oyster (*Crassostrea virginica*) to the Bay has gained momentum as a potential means to enhance N removal and mitigate eutrophication by increasing rates of denitrification [3,4]. Denitrification is the microbially-mediated stepwise reduction of nitrate (NO_3_^-^) and nitrite (NO_2_^-^) to gaseous nitric oxide (NO), nitrous oxide (N_2_O) and dinitrogen (N_2_) [5].

The majority of studies addressing denitrification associated with oysters have primarily focused on whether oysters enhance denitrification in sediments within and adjacent to oyster reefs [6–9]. Oysters may stimulate denitrification by supplying organic carbon (C) and N in the form of biodeposits to denitrifying communities in sediments [4,10,11]. Ammonium (NH_4_^+^) remineralized from oyster biodeposits and excretions can be nitrified to NO_3_^-^, which supports denitrification [3,12]. In addition, the oyster itself can provide a microbial habitat for denitrification (oyster denitrification). Live oysters have been shown to have significantly higher rates of denitrification than sediments [13]. Oyster gut organs in particular, may be hotspots for denitrification, as gut organs of several invertebrates including insects, earthworms, and mussels have shown to exhibit denitrification activity [14–17]. Denitrification in the invertebrate gut is thought to be a result of the anoxic conditions and availability of labile organic carbon provided within the gut environment [15,18]. Oyster shells were also found to have denitrification activity even though the rates were much lower than those measured in live oysters [19]. Shell denitrification may be influenced by factors similar to those impacting sedimentary denitrification. Like oyster reef sediments, the shell microbiome is exposed to increased C and N from biodeposits and excretions, which may enhance denitrification. Both the gut and shell microbiomes are likely important contributors to oyster denitrification, however, no previous studies have identified denitrifying taxa or genes in the oyster microbiome.

Studies investigating the composition of oyster microbiomes are also limited compared to those regarding sediment microbiomes. Previous examinations of oyster microbiomes by cloning and sequencing of 16S rRNA genes, DNA fingerprinting and fluorescent in situ hybridization (FISH) revealed *Proteobacteria* and *Firmicutes* as dominant taxa in different oyster species, but were restrictive in scale or resolution [20–25]. King et al. [26] was one of the first studies using high-throughput next-generation sequencing (NGS) of 16S rRNA gene amplicons to characterize the intestine and stomach microbiome of the eastern oyster. This study showed a dominance of *Mollicutes* or *Planctomycetes* in the oyster stomach, while intestines were found to be more species rich and largely composed of the phyla *Chloroflexi*, *Proteobacteria*, *Verrucomicrobia* and *Planctomycetes* [26]. Follow-up microbiome studies using 16S NGS included further examination of the oyster gut microbiome, as well as microbiomes of oyster gills, mantle and hemolymph [27–30]. For example, in Lokmer et al. [28] higher abundances of *Gammaproteobacteria* were reported in the gut, gill, mantle, and hemolymph microbiomes compared to the surrounding seawater. However, none of the studies to date have attempted to connect the oyster microbiome structure to its function using NGS of 16S rRNA gene amplicons.

Exploring the linkage between the structure and function of microbiomes presents a financial and logistical challenge. Whole-genome shotgun metagenomics offer the ability to identify community structure and functional genes related to metabolic processes in an environment, such as those of microbiomes. Wide-scale, whole-genome metagenomic studies however, are often prohibitively costly and may not be sufficient for large sample sets or for samples where prokaryotic genetic contribution to the metagenome is low [31]. As a result, many microbiome studies rely on much less expensive and accessible 16S rRNA gene based amplicon sequencing, which traditionally has offered little insight into functionality. To address this shortcoming with 16S rRNA gene sequencing, bioinformatic programs, Phylogenetic Investigation of Communities by Reconstruction of Unobserved States (PICRUSt) [32], and more recently PAthway PRediction by phylogenetIC plAcement (paprica) [33], have been developed to infer metabolic pathways from 16S rRNA gene sequences. Several recent studies have used metabolic inference programs to infer microbial metabolisms in marine microbiomes such as those of macrobiota biofilms [34], sponges [35], and corals [36]. Some key differences in the programs are in the assignment of pathways and user flexibility. PICRUSt uses ancestral state reconstruction to infer the probable metabolism (according to the KEGG ontology [37]) of extant Greengenes operational taxonomic units (OTUs) [38]. In comparison, paprica describes community structure through phylogenetic placement with pplacer [39] onto a reference tree created from all completed genome in Genbank [40]. Paprica then uses a pre-computed database to assign genomic features (including genes and metabolic pathways via the MetaCyc ontology [41]). Paprica is designed to maximize user flexibility and has options for adding reference draft genomes and customizing the enzyme commission (EC) numbers associated with reference genomes.

We combined a customized database of genomes and denitrification genes with the paprica program to link the oyster digestive gland (gut), shell, and reef sediment microbiome structures to denitrification by characterizing the composition of microbiomes and identifying potential denitrifiers from 16S rRNA amplicon sequences. Our main objectives were to (1) compare the oyster microbiomes’ taxonomic classifications determined by paprica and other taxonomic databases, (2) examine the structure and diversity of the oyster microbiomes using a taxonomically independent OTU analysis, and (3) connect the oyster microbiome to rates of denitrification by comparing the relative abundances and composition of denitrification genes in each microbiome to measured N_2_ fluxes. A customized paprica database was constructed with dissimilatory nitrite reductase genes (*nir*S and *nir*K), and nitrous oxide reductase gene (*nos*Z) identified from completed or draft genomes. *Nir*S and *nir*K encode enzymes responsible for the reduction of nitrite (NO_2_^-^) to nitric oxide (NO), while *nos*Z encodes for enzyme in the reduction of nitrous oxide (N_2_O) to nitrogen gas (N_2_) in the denitrification pathway. The *nos*Z gene classification was further divided into two separate clades; clades I (*nos*ZI) and II (*nos*ZII). Gene clades *nos*ZI and *nos*ZII differ based on variations in signaling peptides, phylogeny [42], and responses to environmental conditions [43,44]. Continuous flow experiments were performed with live oysters, empty shells, and reef sediments to measure the associated denitrification activity.

## Materials and methods

### Sample collection and flow-through experiment

Triplicate samples of live oysters, pairs of empty oyster shells, and intertidal surficial sediment cores taken within oyster reefs [45] were collected on 7 July 2013 at low tide from Hoop Hole Creek (Latitude 34.706483, Longitude 76.751931), a tidal creek located in Atlantic Beach, NC, and immediately transported to the University of North Carolina Institute of Marine Sciences (UNC IMS). Oysters samples were acquired according to conditions detailed in UNC IMS’s research collection permit from NC Division of Marine Fisheries. Temperature, salinity, dissolved oxygen (DO) were measured using a YSI water quality sonde (YSI, Inc.). Water was filtered through Whatman GF/F filters (25 mm diameter, 0.7 l m nominal pore size) and the filtrate was analyzed with a Lachat Quick-Chem 8000 automated ion analyzer for NO_3_^-^.

Sediment cores were left in a water bath overnight with continuous aeration with air stones. Oysters and shells were stored overnight in raceway flumes and then added to individual cores and capped the following morning. Continuous, flow-through core incubation experiments to measure N_2_ fluxes were conducted under dark conditions in an environmental chamber held at constant site water temperatures using each of the collected samples. The treatments consisted of: (1) live oyster, (2) oyster shells only, and (3) sediment. Samples from the bypass line (flowed directly from reservoir to 5ml ground glass vial) and each core’s outflow were collected following the acclimation period. Inflow water and outflow water leaving the cores were analyzed for dissolved N_2_, O_2_ and Ar using a Balzers Prisma QME 200 quadruple mass spectrometer [46]. Concentrations of O_2_ and N_2_ were determined using the ratio with Ar [46,47]. Following the experiment, oysters, oyster shells, and 50 mL of sediment from the cores were frozen and shipped to the Virginia Institute of Marine Science, where they were stored at -80°C.

Whole oysters were partially thawed at room temperature for approximately 30 minutes before dissection. Dissections were carried out using sterile scalpel blades. Digestive glands were carefully excised, transferred to 2.0 mL microcentrifuge tubes, and frozen at -80°C. Following dissection, the remaining oyster tissue was removed from its shell, and the interior of the shell was scrubbed with 75% ethanol. Oyster shells from live oysters (shell (live)) and paired oyster shells collected from the reef (shell (only)) were crushed into roughly 0.5–5.0 mm sized pieces using sterilized hammers to homogenize the exterior shell biofilm. Shell fragments were then transferred to 50 mL falcon tubes, and frozen at -80°C.

### DNA extraction and amplification

DNA was extracted from 0.25–0.30 grams of digestive gland using the Qiagen DNA stool mini kit (Qiagen, Hilden, Germany) following the pathogen detection protocol. Shell (0.40–0.60 grams) and sediment (0.50–0.75 grams) extractions were conducted using MoBIO Powersoil extraction kits (Mo-Bio Laboratories, Inc., Carlsbad, CA) following the manufacture’s protocol. As a result of variation in the source material, different kits were used to extract DNA from the oyster digestive gland and the oyster shell or reef sediment in order to optimize DNA quality and DNA yield for PCR and sequencing efficiency. While this may introduce some bias, these biases tend to have a minimal impact on 16S NGS microbiome studies [48]. Overall, 12 DNA samples were extracted: triplicate DNA samples from (1) oyster digestive gland, (2) shell from live oysters, (3) collected (empty) paired shells, and (4) oyster sediment.

Initial amplification of the targeted hypervariable V4 region of the 16S rRNA gene was performed on extracted DNA using forward primer 515F and modified, barcoded reverse primer 806R [49], adapted for use with the Ion Torrent Personal Genome Machine (PGM). The basic manufacturer’s PCR protocol was used with Taq DNA Polymerase (Invitrogen, Carlsbad, CA) to create a PCR master mix with the following modification: 1 mM dNTP mixture was used in place of 10 mM for a final concentration of 0.02 mM dNTP. Thermal cycling conditions consisted of an initial denaturation step at 94°C for 3 min, followed by 30 cycles of 94°C for 1 min, 54°C for 1 min, 68°C for 2 min. A final elongation step of 68°C for 10 min was added to ensure complete amplification. The amplified products were gene cleaned using the UltraClean GelSpin DNA Purification Kit (Mo-Bio Bio Laboratories, Inc., Carlsbad, CA). The resulting amplicon libraries were then used as templates for sequencing with the Ion S5 platform following the manufacture’s instruction (Thermo Fisher Scientific, Waltham, MA). Sequences generated in this study may be downloaded from the NCBI Sequence Read Archive, accession number SRP106715.

### Bioinformatic analyses

An overview of the bioinformatic pipeline used for the 16S rRNA based microbiome analyses is shown in supplementary materials (S1 Fig). Removal of barcodes and primers from raw sequences and trimming of sequence length were conducted using the Ribosomal Database Project (RDP) pipeline initial process [50] (http://rdp.cme.msu.edu) with a minimum quality score of 20, minimum length of 200 bases, and a maximum length of 500. Mothur v1.35.1 [51] was used to further trim sequences against the SILVA v123 [52] alignment template, precluster, and screen for chimeric sequences using the uchime *denovo* program [53]. Unknown taxa, mitochondria, chloroplast, archaea, and eukaryotic sequences were removed from analysis using SILVA v123 reference taxonomy and the Wang classification method [54] with an 80% minimum identity. Archaea were excluded from this analysis due to their low abundances; archaea comprised <1.0% of the total overall sequencing reads and made up <3.8% of the reads in any one sample. Further analyses focused on high quality bacterial sequences only.

Phylotype analyses using Mothur were conducted on high quality, trimmed bacterial sequences to determine the taxonomical composition of oyster digestive gland, oyster shell, and oyster reef sediment microbiomes. Sequences were classified with SILVA v123, Greengenes v13_5, or RDP v14 reference taxonomy databases using the Wang classification method described previously. For all phylotype analyses, resulting taxonomic relative abundances from triplicate microbiome samples were averaged together, with oyster shells from live oysters (shell (live)) and collected paired shells (shell (only)) combined together to from the oyster shell microbiome. In addition, an operational taxonomic (OTU) analysis was conducted on the microbiome sequences to assess microbiome diversity. Sequences were clustered into OTUs based on a 97% identity using the average neighbor clustering algorithm. To remove sampling intensity error and normalize samples, individual sample reads were randomly subsampled to the lowest number of reads found in the sample data set (n = 66,687). All diversity metrics are based on microbiome averages. For diversity metrics, both shell (live) and shell (only) treatments described previously were combined to form the shell microbiome; for principle coordinate analysis (PCoA), shell (live) and shell (only) microbiomes were analyzed separately to determine shell microbiome structure similarity.

To conduct phylotype and denitrification gene inference analyses using paprica, a customized paprica database was constructed with 5,445 complete and 222 draft bacterial genomes (S1 and S2 Tables). High quality draft genomes, where available, were selected for inclusion in the database based on their relevance to oyster microbiome taxonomical structures determined by the Silva, Greengenes, and RDP phylotype analyses. All draft genomes were downloaded from GenBank (https://www.ncbi.nlm.nih.gov/genbank/). Each individual genome was curated for the presence of *nir*S, *nir*K, and *nos*Z genes using either the KEGG database for completed genomes or gene annotations for draft genomes. Construction of the paprica reference database and inclusion of the gene-specific inferences were conducted following the instructions found on the developer’s website (http://www.polarmicrobes.org/building-the-paprica-database/). Phylotype and gene inference analyses were performed by first aligning the quality controlled query reads to the reference alignment with Infernal, then placing them on the phylogenetic reference tree with pplacer [39]. Taxonomical classification and gene inferences were based on edge placement and consensus identity with either internal or terminal nodes as described in Bowman and Ducklow [33]. Resulting abundances from paprica were given as either values normalized to 16S rRNA gene copy number or as uncorrected values. Normalized values were calculated as the measured abundance divided by the number of 16S rRNA gene copies predicted for each taxon. Uncorrected values were used for the phylotype analysis to perform an equivalent comparison with the Mothur phylotype analyses, while normalized values were used with gene abundances to better capture potential denitrifiers. Distinctions between *nos*ZI and *nos*ZII gene abundances and taxonomic classification were based on edge taxonomies only.

### Quantitative PCR

Quantitative PCR (qPCR) assays were performed on oyster and sediment samples to determine the relative abundance of *nos*ZI genes. Relative abundances of *nos*ZI genes in each sample were calculated using the ratio of *nos*ZI abundance to the abundance of 16S rRNA genes. Gene abundances for *nos*ZI and 16S rRNA were determined using the 6 Flex Real-Time PCR system (Thermo Fisher Scientific, Waltham, MA). 16S rRNA gene qPCR assays were carried out in a volume of 20 μL consisting of 10 μL of 2X SYBR green based GoTaq qPCR Master Mix, 0.05 μL CXR reference dye (Promega Corporation, Madison, WI), 0.01 mg/mL BSA (Promega Corporation, Madison, WI), 0.5 μM each of 16S rRNA specific primers EU341F and 685R targeting hypervariable regions V3, and 1 uL of template DNA. Thermal cycling conditions consisted of an initial denaturing step at 95°C for 10 min, followed by 30 cycles of 95°C for 15 s, 55°C for 30 s, and 72°C for 30 s with fluorescence detection. Quantification of *nos*ZI was performed using the same reaction volumes and components described for 16S, with *nos*ZI specific primers nosZ1F and nosZ1R [55]. Thermal cycling conditions for *nos*ZI qPCR were the same as 16S with the exception that total cycle number was increased to 50 cycles, elongation step at 55°C was increased to 45 s, and additional step at 80°C with fluorescence detection was added. All reactions were performed on 96-well plates with duplicate negative controls and standards. Standards were prepared by serially diluting plasmids carrying either the 16S or *nos*ZI gene and quantified with the Agilent 220 TapeStation System (Agilent Technologies, Santa Clara, CA). Standard curves and gel electrophoresis were used to confirm reaction specificity.

### Statistical analyses

For all results, variation within each microbiome is reported as the standard deviation. Diversity statistics including coverage, Chao I, and Shannon were conducted using the summary.single command in Mothur. A principle coordinate analysis (PCoA) and the Adonis function for Permanova (non-parametric permutational multivariate analysis of variance; Anderson [56]) using Bray-Curtis dissimilarity were performed on OTU distributions with the Phyloseq package [57] in R (version 3.1, https://wwww.R-project.org). Flux data was assessed for normality using the qqplot function and Shapiro-Wilk normality test (p < 0.05). One-way analysis of variance (ANOVA) and a post-hoc Tukey honest significant difference (HSD) tests were performed on flux measurements to test for significant differences. A one-tailed, paired t-test was used to determine differences between *nos*ZI and *nos*ZII within the shell and sediment microbiomes, and a one-tailed, Welch’s t-test was used to compare gene abundances between the shell and sediment microbiomes. For comparisons between the oyster, shell, and sediment microbiomes, relative abundances of the digestive gland microbiome and shell (live) microbiome were combined to form the oyster microbiome. A simple linear regression analysis was conducted to compare the relative abundances of *nos*ZI measured by qPCR and the uncorrected *nos*ZI gene abundances predicted by paprica; uncorrected paprica values were used so that equivalent comparisons between gene abundances and qPCR relative abundances could be made. Unless otherwise stated all statistics were conducted in R and significance was based on p < 0.05.

## Results

### Phylotype comparison of microbiomes

**Fig 1:**
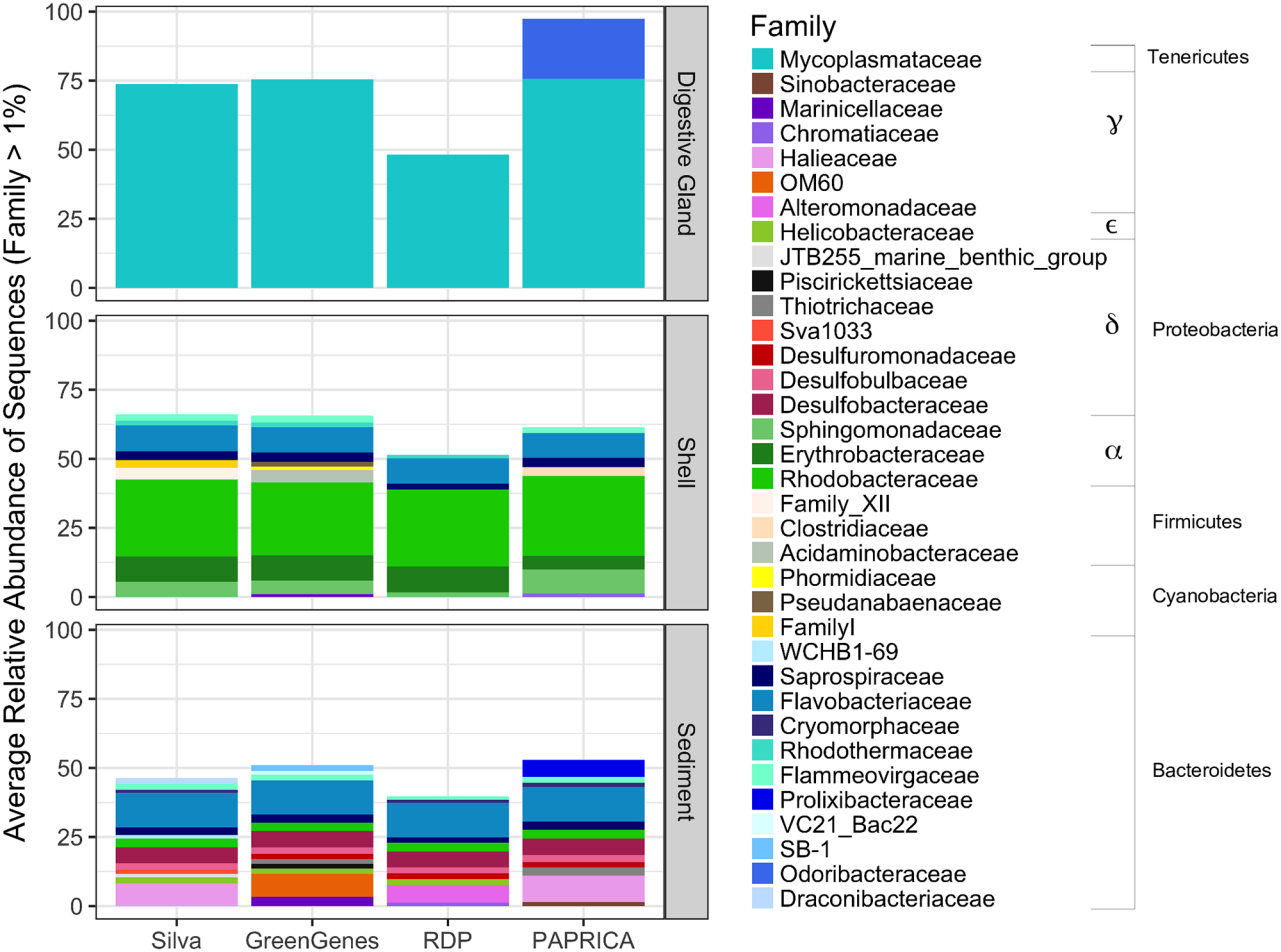
Average relative abundances of bacterial families in the oyster-related microbiomes, classified by different reference databases. Families with ≥ 1% relative abundance in samples are shown. Shell microbiome consists of shell (live) and shell (only) treatments.

A total of 982,504 trimmed, high quality 16S rRNA gene sequences were obtained from the oyster and sediment microbiome samples. Sequencing depth averages for each microbiome were 85,640 ± 1.5x10^4^ for oyster digestive gland, 86,745 ± 1.6x10^4^ for shell, and 68,378 ± 1.5x10^3^ for sediment. Among the 4 databases, paprica classified the greatest number of sequences at the family level (85.4 ± 9.8%), followed by Silva (76.5 ± 18.9%), Greengenes (75.8 ± 18.5%), and RDP (57.2 ± 18.7%). All four databases showed an overall similar pattern at the family classification level for the average relative abundance of sequences ≥1% (Fig 1). With the exception of one shell in the shell (only) treatment having a slightly different profile (S2 Fig), phylotype comparisons between the shell (live) and shell (only) microbiomes were similar in taxonomy and relative abundance, and were thus combined together to form the shell microbiome. Of the oyster-related microbiomes, the sediment microbiome showed the greatest number of families (n = 12.5 ± 1.7) and the lowest percent of sequences identified (47.7 ± 6.7%), the oyster digestive gland microbiome showed the lowest number of families (n = 1.3 ± 0.5) and the highest number of sequences identified (73.1 ± 24.5%), and the oyster shell microbiome fell somewhere in the middle (n = 8.8 ± 2.5; 59.7 ± 7.7%) (Fig 1). Each of the four databases consistently identified family *Mycoplasmataceae* from phylum *Tenericutes* as the dominant family in the digestive gland microbiome. Paprica was the only method to also include the classification of *Odoribacteraceae* as another dominant family member in the digestive gland microbiome. Within the oyster shell microbiome, all four databases showed a dominance of families *Sphingomonadaceae*, *Erythrobacteraceae*, and *Rhodobacteraceae* from phylum *Proteobacteria*, and *Flammeovirgaceae*, *Flavobacteriaceae*, and *Saprospiraceae* from phylum *Bacteroidetes*. *Desulfobacteraceae* and *Rhodobacteraceae* from phylum *Proteobacteria*, and *Flavobacteriaceae* and *Saprospiraceae* from phylum *Bacteroidetes*, were the dominant families consistently identified in the sediment microbiomes across all four databases. The greatest variation among the databases in the classification of families occurred in paprica’s identification of sequences from phylum *Bacteroidetes* and Greengenes’s identification of sequences from phylum *Proteobacteria*. However, at the phylum level, identification of sequences for each phylum was relatively consistent among the four databases.

### Diversity comparison of microbiomes using OTU analysis

**Fig 2:**
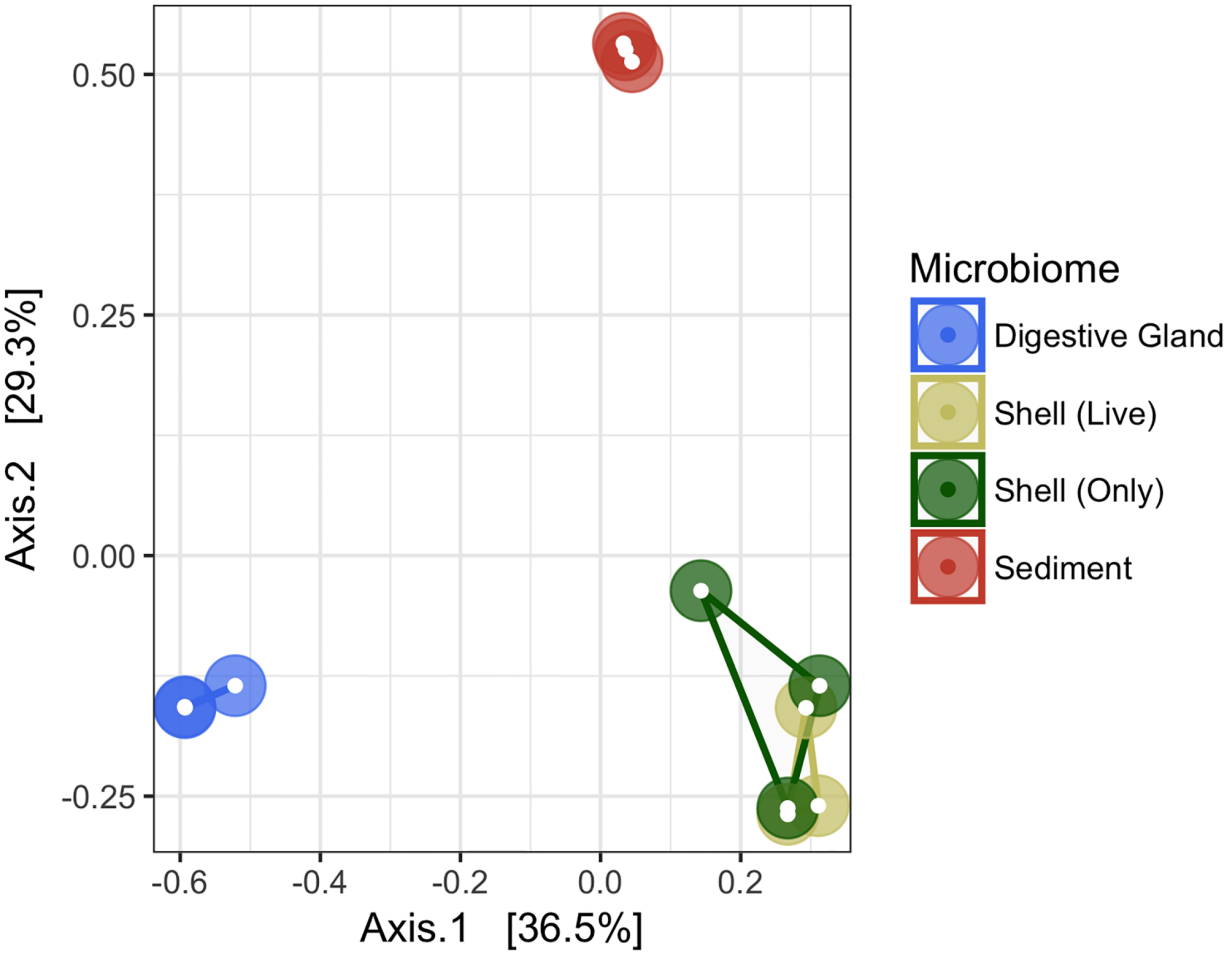
Principal coordinate analysis (PCoA) of oyster-related microbiomes. PCoA based on 16S rRNA gene sequences using Bray-Curtis similarity matrix.

All 12 microbiome samples were subsampled to 66,687 sequences to conduct an OTU diversity analysis (Table 1 and Fig 2). Average coverage of sequences ranged from 89.1 ± 0.9% in the sediment microbiome to 99.6 ± 0.0% in the oyster digestive gland. Significant differences among the microbiomes were detected with Permanova (*F*_2,11_ = 8.19, p = 0.001) and demonstrated using PCoA (Fig 2), which explained 65.8% of the variation found. The oyster digestive gland, shell, and sediment samples, formed distinct microbiomes, clustering separately based on sample type. The greatest dissimilarity occurred between the oyster digestive gland and the sediment microbiome. There were no differences between the shell microbiomes, whether the shell came from a live oyster or a discarded, empty shell. Similar trends were found among the microbiomes regarding Chao I richness, Shannon diversity, and OTU abundances (Table 1). Sediment microbiomes had the highest level of diversity and richness than all other microbiomes (Chao 1 = 32,035 ± 1.8x10^3^, Shannon = 6.8 ± 0.2), and an average OTU abundance of 10,489 ± 9.3x10^2^. Shell microbiome had moderate diversity and richness (Chao 1 = 18,025 ± 3.2x10^3^, Shannon = 5.7 ± 0.5) with an average OTU abundance of 6,264 ± 1.5x10^3^, and the oyster digestive glands had the lowest levels of diversity and richness (Chao 1 = 1,234 ± 1.7x10^2^, Shannon = 1.2 ± 0.1) with an average OTU abundance of 525 ± 4.1x10^1^.

**Table 1:** Summary statistics of 16S rRNA gene amplicon sequencing for oyster-related microbiomes.

Sample	No. of OTUs^a^	Coverage (%)	Chao Index	Shannon Diversity
Digestive Gland 1	477	1.00	1038.17	1.06
Digestive Gland 2	545	1.00	1292.67	1.10
Digestive Gland 2	552	1.00	1372.33	1.33
Shell (Live) 1	6,508	0.93	18777.19	5.46
Shell (Live) 2	7,491	0.93	22004.59	6.31
Shell (Live) 3	6,387	0.94	19435.59	5.90
Shell (Only) 1	7,027	0.93	18966.56	6.24
Shell (Only) 2	5,616	0.94	16288.26	5.37
Shell (Only) 3	4,555	0.96	12681.37	5.19
Sediment 1	10,946	0.89	33237.59	6.88
Sediment 2	9,417	0.90	29882.63	6.57
Sediment 3	11,106	0.89	32986.13	7.00

All metrics are based on subsamples of n = 66,687.

^a^ OTUs are based on 97% sequence identity using Mothur’s average neighbor clustering algorithm

### Microbiome denitrification gene inferences with the paprica database

**Fig 3:**
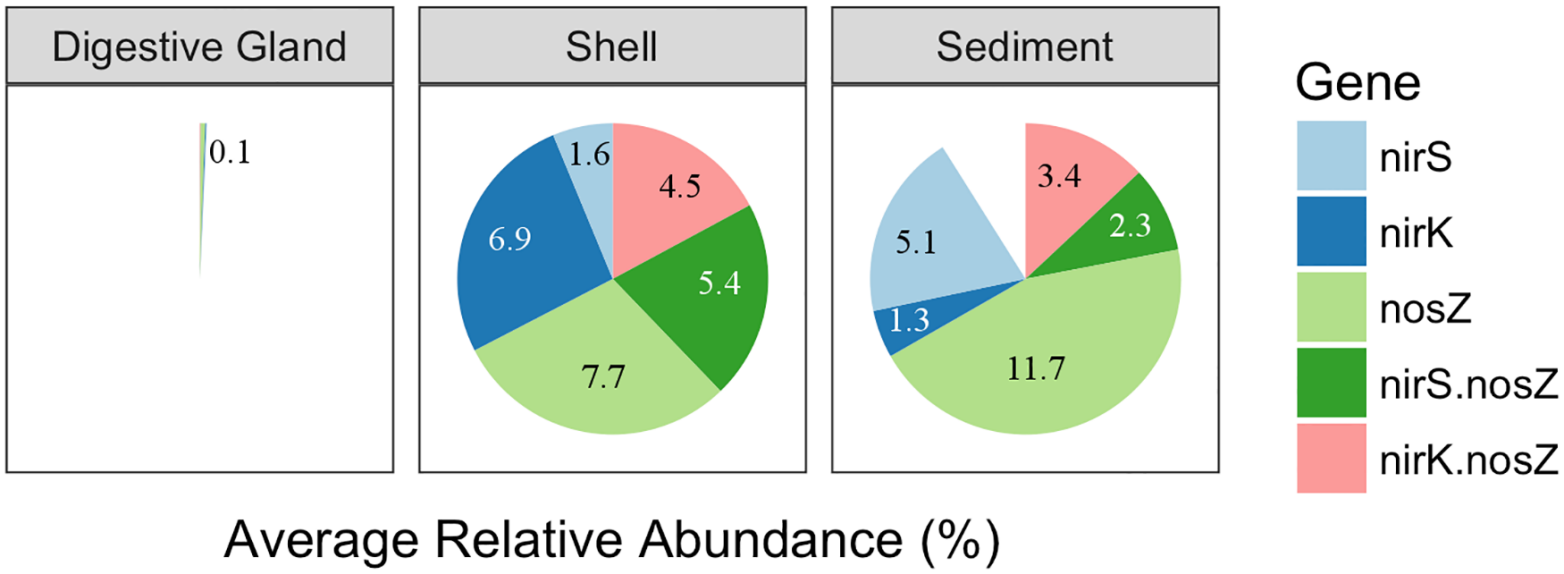
Predicted average relative abundances of denitrification genes by paprica for oyster-related microbiomes. Shell microbiome includes shell (live) and shell (only) treatments. Each full circle represents a relative abundance of 26.1%.

**Fig 4:**
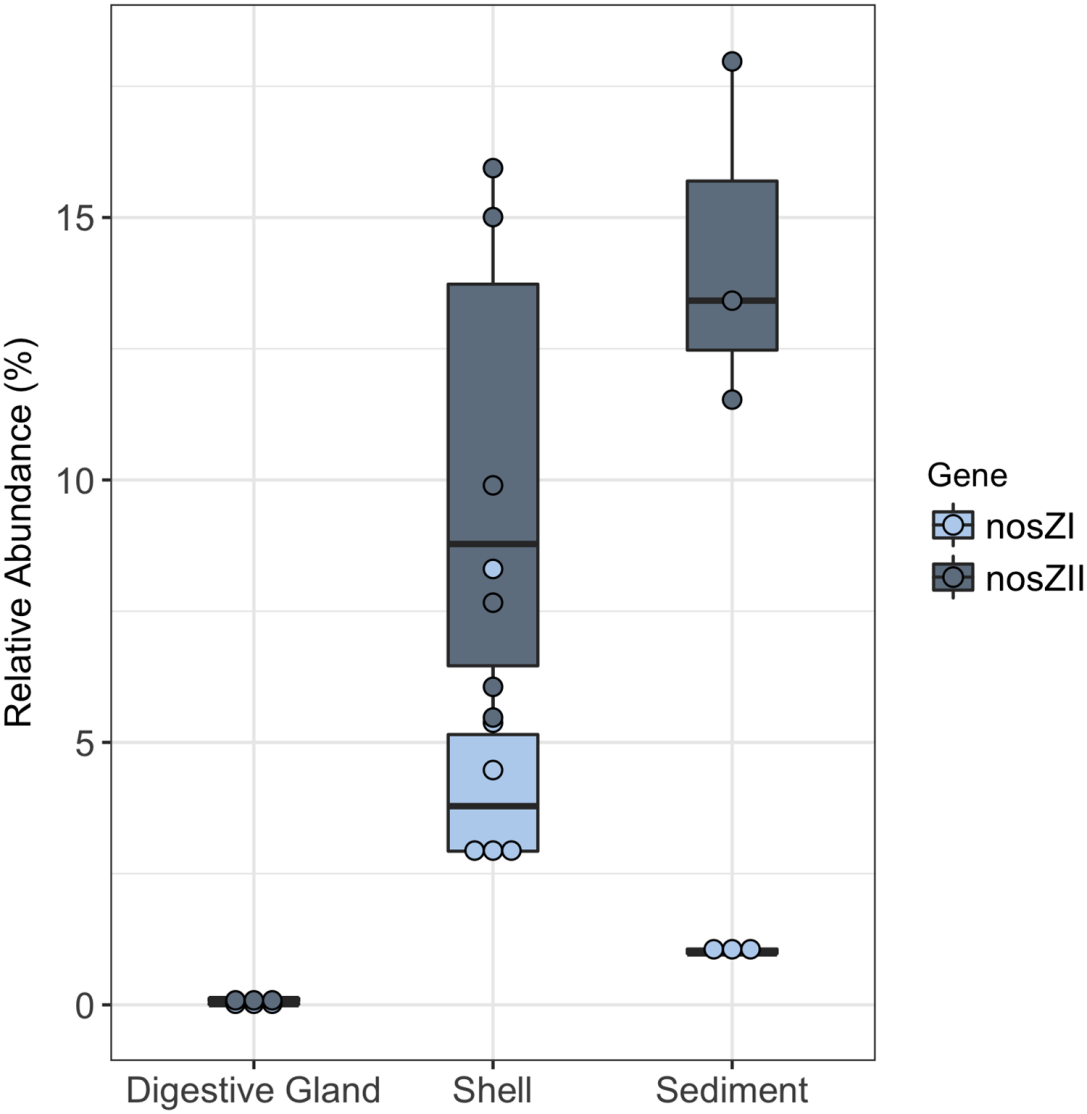
Predicted relative abundances of genes *nos*ZI and *nos*ZII by paprica in oyster-related microbiomes. Shell microbiome includes both shell (live) and shell (only) treatments.

The sediment and shell microbiomes had an inferred average relative abundance of 23.8 ± 2.8% and 26.1 ± 3.0%, respectively, of denitrification genes (Fig 3). The digestive gland microbiome was comprised of a ≤ 0.1% relative abundance of denitrification genes. The greatest differences among the microbiomes were found in the relative abundances of the *nir*K, *nir*S, or *nos*Z genes only. Combined, organisms carrying one of these genes were more dominant than organisms carrying both *nir*S and *nos*Z or *nir*K and *nos*Z genes. Between the shell and sediment microbiomes, the shell microbiome had a significantly higher relative abundance of bacteria carrying the *nir*K only gene (unpaired t-test *t*_5_ = 6.48, p = 2.6x10^-5^), while the sediment had a significantly higher abundance of the *nir*S only (unpaired t-test *t*_7_ = 8.75, p = 2.6x10^-5^) and a higher, but not significant, abundance of *nosZ* only (unpaired t-test *t*_7_ = 2.74, p>0.05) genes. Among the microbiomes, the average relative abundance of organisms carrying *nos*ZII gene was overall higher than those carrying the *nos*ZI gene (Fig 4). In the sediment microbiome, this difference was significant (paired t-test *t* = 7.14, p = 9.5x10^-3^), but it was not significant in the shell or digestive gland microbiomes. Taxonomically, *nosZ*I bacteria were primarily from class *Alphaproteobacteria*, while *nosZ*II bacteria were from classes *Cytophygia* and *Flavobacteriia* in the shell, and *Gammaproteobacteria*, *Cytophygia*, and *Flavobacteriia* in the sediments (S3 Fig).

### N_2_ flux experiments

**Fig 5:**
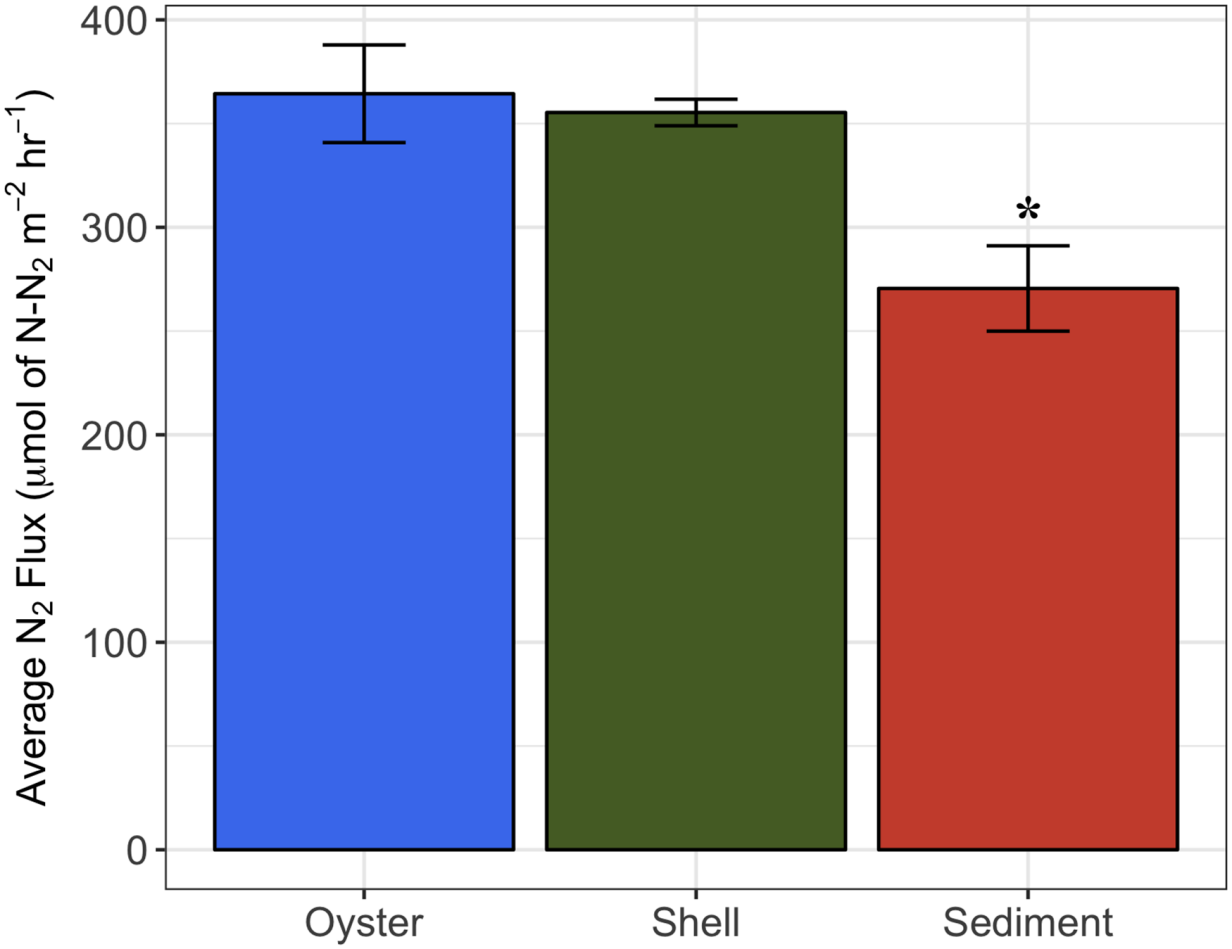
N_2_ flux measurements from oysters, shell only, and sediment treatments using a continuous flow through design. For each treatment n = 3 and error bars represent ± s.d. (*) significance p < 0.05.

Site water physical and chemical parameters used in the flux experiments were as follows: 30°C temperature, 30 ppt salinity, 6.8 mg/L dissolved oxygen (DO), and 0.51 μmol N/L NO_3_^-^. Live oyster cores had the highest average flux of N_2_ at 364.4 ± 23.5 μmol N-N_2_ m^-2^ h^-1^, followed by the shell only cores at 355.3 ± 6.4 μmol N-N_2_ m^-2^ h^-1^, and sediment cores with the lowest at 270.5 ± 20.1 μmol N-N_2_ m^-2^ h^-1^ (Fig 5). There were no significant differences in the N_2_ fluxes between the live oyster and shell, but both were significantly higher than the sediment cores (ANOVA, *F*_2,6_ = 23.7, p = 1.4x10^-3^; Tukey HSD, p < 0.05).

### Microbiome nosZI gene inference comparison to flux measurements and qPCR

**Fig 6:**
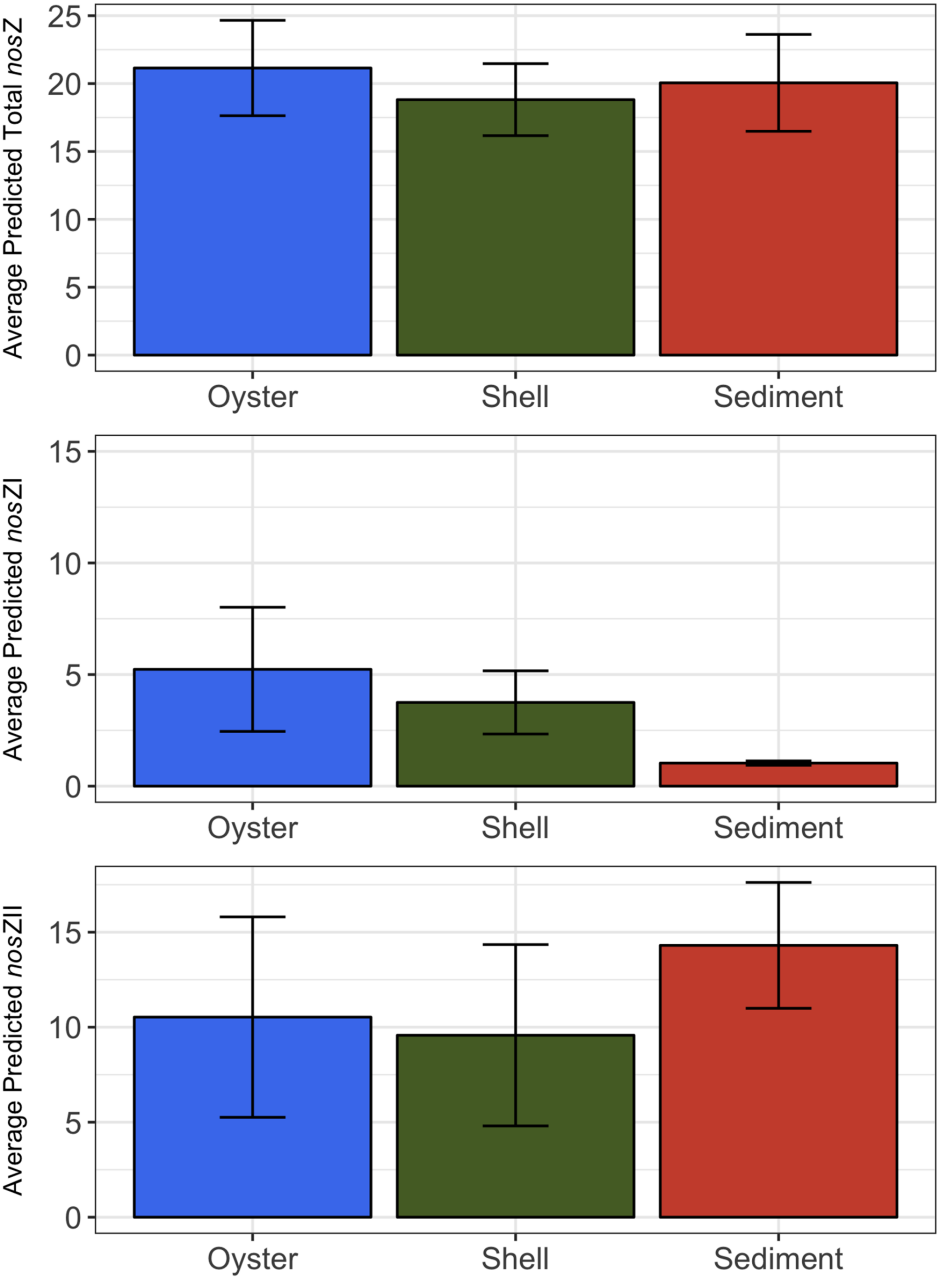
Average predicted relative abundances of total (A) *nos*Z, (B) *nos*ZI, and (C) *nos*ZII by paprica for oyster-related microbiomes. Digestive gland combined with shell (live) to form oyster microbiome. Shell (only) forms shell microbiome. For each treatment n = 3 and error bars represent ± s.d.

**Fig 7:**
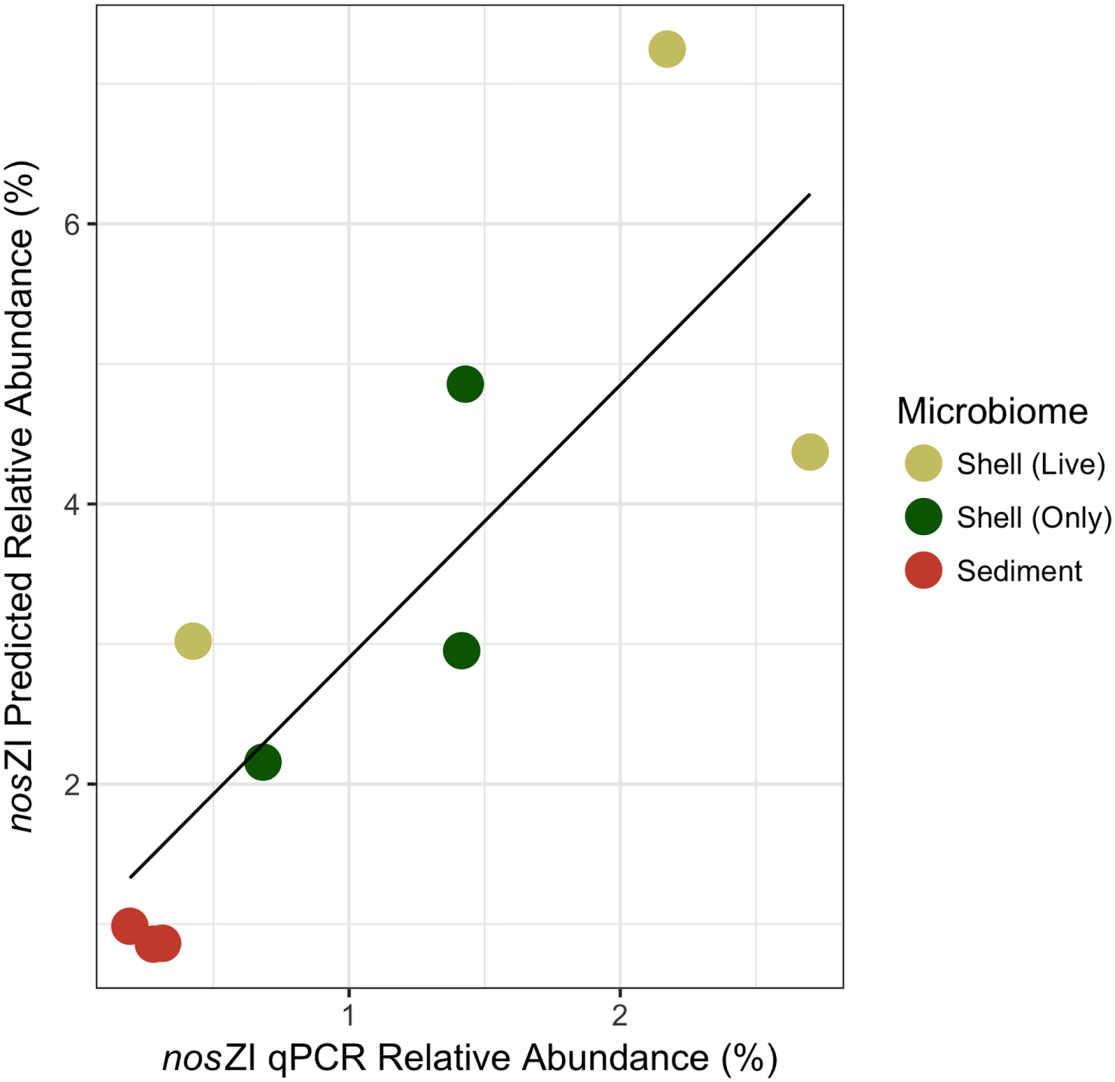
Linear regression comparing predicted and quantified relative abundances of *nos*ZI genes for shell (live), shell (only) and sediment microbiomes. Predicted relative abundances based on paprica inferred *nos*ZI gene abundances relative to 16S gene abundances. Quantified relative abundances based on qPCR of *nos*ZI gene copy numbers relative to 16S gene copy numbers.

The rates of N_2_ fluxes followed a similar trend to the average relative abundance of *nos*ZI genes inferred in oyster, shell, and sediment microbiomes (Figs 5 and 6B). Oysters and shells had similarly high N_2_ flux rates and *nos*ZI genes, while sediment samples had lower rates of N_2_ flux and lower abundances of *nosZ*I genes. This trend was not found in the average relative abundance of the *nos*ZII genes or in overall *nosZ* gene abundance (Figs 5, 6A and 6C). A significant, positive linear correlation was determined between the copy number of *nos*ZI genes quantified in the shell and sediment microbiomes by qPCR and the relative abundance of *nos*ZI genes inferred from paprica (*F*_*1*,7_ = 14.7, p = 6.0x10^-3^, *R*^2^ = 0.68) (Fig 7). Predicted values were on average 3.5 ± 1.7% x higher than those determined by qPCR. Copy numbers of *nos*ZI genes from oyster digestive gland microbiome samples were below detection level, and thus were excluded from the regression analysis.

## Discussion

Paprica’s taxonomical classification of the oyster digestive gland, oyster shell and sediment microbiomes was comparable to other reference databases regarding the pattern of dominant families found within each microbiome (Fig 1). All four phylotype analyses in this study showed *Mycoplasmataceae*, from phylum *Tenericutes*, to clearly be dominant in the oyster digestive gland. While studies on oyster gut-related microbiomes are relatively small in number, several studies including Green and Barnes [22], Lokmer et al. [28], and King et al. [26] found *Mycoplasma* to be highly abundant in digestive glands of Sydney rock oysters (*Saccostrea glomerata*), gut tissues of pacific oysters (*Crassostrea gigas*), and stomachs of eastern oysters, respectively. Even less is known about the oyster shell microbiome. While no known studies to date have examined the structure of the oyster shell microbiome, a related study conducted on mussel (*Mytilus californianus*) shell surface communities found in the Pacific Northwest showed *Gammaproteobacteria* to be the dominant class [34]. In comparison, our study found *Alphaproteobacteria* to be the dominant class in the oyster shell microbiome, while *Gammaproteobacteria* (and *Deltaproteobacteria*) were more dominant in the sediment microbiome. *Alphaproteobacteria*, in particular *Roseobacter* from family *Rhodobacteraceae*, have been shown to rapidly colonize surfaces in Atlantic temperate waters and may produce antibacterial components, preventing other bacteria from growing [58]. This may explain our findings in the shell microbiomes, which were dominated by family *Rhodobacteraceae*. In the sediment, *Gamma-* and *Deltaproteobacteria* have been shown to be highly abundant in surface sediments [59–61], which is consistent with our findings. In addition to *Proteobacteria*, *Bacteroidetes* was another dominant phlyum in both shell and sediment microbiomes. *Bacteroidetes* are common in the marine environment [62], and thus likely to be present in marine samples exposed to the environment.

Diversity of the microbiome determined by the paprica phylotype analyses was compared with a taxonomically independent OTU analysis performed by the Mothur program. The PCoA analyses (Fig 2) verified that the oyster digestive gland, shell, and sediment microbiomes were structurally different from each other, but also that the variation within each microbiome was relatively low. Interestingly, the microbiome structure between shells from live oysters vs. those from shells only was highly similar. Shells used in the shell only treatment grew and were collected on the same oyster reef from which the whole oysters were collected. Further studies would need to be conducted to see if mere proximity to an oyster or oyster reef influences the shell microbiome, or if once an oyster’s shell microbiome is established, the microbiome remains after the animal has expired. The high similarity between samples within each microbiome provided a realistic ability to measure differences between the microbiomes despite the small number of samples analyzed, and a sufficient justification to assess microbiome structure based on pooled averages. Similarly, the diversity and richness patterns (Table 1) determined by the OTU analysis followed the same pattern as the taxonomical diversity demonstrated in the phylotype analyses (Fig 1). In the sediments, for example, high diversity and richness corresponded to a greater number of taxonomical families and a more even distribution of those families. Additionally, coverage of the microbiomes was determined to be ≥ 89% in the most OTU rich samples (Table 1), indicating that the microbiome structure was adequately sampled, and inferences drawn from the microbiomes were representative of the community structure. All of these factors combined demonstrated that the taxonomical classifications determined by the paprica database accurately and thoroughly described the microbiome structures. This allowed for reasonable confidence in using the modified paprica database to infer the abundance and distribution of denitrification related genes in the oyster digestive gland, shell, and sediment microbiomes.

Despite having different taxonomical profiles (Fig 1) and distinct microbiome structures (Fig 2), The average relative % abundances of bacteria carrying *nir*S, *nir*K, *nos*Z, or a combination of those genes, were similar in the shell and sediments, making up between ~ 23–26% of the overall community (Fig 3). This suggests the abundance and distribution of denitrifying bacteria carrying these genes may be conserved between microbiomes. However, this pattern changed with respect to individual gene abundances. Both shell and sediment microbiomes had relatively similar overall abundances of *nir* only genes, yet *nir*K only was significantly more abundant in the shell microbiome, while *nir*S only was significantly more abundant in the sediment microbiome. In estuarine systems, *nir*S has been generally shown to be more abundant than *nir*K [63,64]. However, *nir*K has been shown to be dominant in environments associated with animal hosts [43] and in zones of high oxygen and pH fluctuation, like those found in microbial mats [65]. The higher abundance of *nir*K carrying bacteria versus *nir*S in oyster shells may be evidence of the shell microbiome’s (current or past) connection to an oyster host, or a result of the potentially more oxic environment provided by the shell surface, compared to the marine sediment. Also interesting, is that in both microbiomes the predicted relative abundances of complete denitrifiers, those carrying the *nir* and *nos* genes together, were less than those carrying either the *nir* or *nos* genes separately. This indicates that the complete transformation of NO_2_^-^ to N_2_ in of these microbiomes may be highly modular and dependent on community interaction and not individual denitrifiers.

Regarding *nos*Z gene abundances, all oyster-related microbiomes, showed the predicted relative abundance of *nos*ZII bacteria were higher than *nos*ZI carriers (Fig 4). This is consistent with other studies that have shown *nos*ZII denitrifiers to be dominant over *nos*ZI denitrifiers in a variety of different environments [42,66]. Microbes with the *nos*ZII gene have been shown to be more taxonomically and ecophysiologically diverse than those with *nos*ZI genes [67]. This was evident in the shell and sediment microbiomes in our study. Among the shell and sediment microbiomes, the primary driver of *nosZ*I abundances belonged to bacteria from a single class, *Alphaproteobacteria*, while *nosZ*II abundances were mainly driven by bacteria belonging to classes *Cytophygia*, and *Flavobacteriia* in the shell and *Gammaproteobacteria*, *Cytophygia*, and *Flavobacteriia* in the sediments (S3 Fig). Additionally, among all three microbiomes, as diversity increases in the microbiome, the differences between *nosZ*I and *nosZ*II abundances became much greater. This may suggest that *nos*ZII abundances may be positively linked to microbiome diversity.

Net N_2_ production measured by flux experiments in this study determined that oysters and oyster shells had a significantly higher net production of N_2_ compared to sediments (Fig 5). Comparisons between oyster nitrogen cycling studies are complicated by the unit at which studies are conducted (whole reef, sediments, oysters, shells), the type of incubation (flow through vs. batch), and the setting of the oysters (natural reef, constructed reef, aquaculture). Despite all of these distinctions in oyster nitrogen cycling studies, we found the results from this study to be largely similar to previous research. Sediment N_2_ production in this study was in line with summer values for oyster reef sediments in nearby reefs [13,45]. N_2_ production by oysters alone were in agreement with the results in Smyth et al. [13], which also found live oysters to have higher net N_2_ fluxes than tidal flat sediments. Shell only rates were lower than those in Caffrey et al. [19].

Predicted relative abundances of *nos*Z (combined *nos*ZI and *nos*ZII), the gene responsible for transforming N_2_O to N_2_ (Fig 6A) and thus expected to be highest in microbiomes with the greatest denitrification, showed the opposite trend. The highest relative abundances of *nos*Z genes were found in the reef sediments with the lowest N_2_ fluxes, while the lowest relative abundances of *nos*Z genes were found in the oyster shell (only) and in the oyster (combination of shell (live) and oyster digestive gland) with the highest N_2_ fluxes. This may be a result of DNA-based gene abundances failing to correlate with gene expression. However, when *nos*ZI and II are analyzed separately, a pattern similar to the flux rates emerges with *nos*ZI abundance (Fig 6B and 6C). A significant positive correlation between *nos*ZI abundance measured by qPCR and the predicted relative abundances of *nos*ZI verified that as denitrification flux rates increased, so did the abundances of *nos*ZI (Fig 7). This pattern was not seen in the more dominant *nos*ZII gene abundances, suggesting that *nos*ZI carriers may be more important to denitrification in oyster microbiomes than *nos*ZII carriers. As mentioned previously, many organisms may carry the *nos*Z gene, but do not necessarily express the *nos*Z gene. Organisms carrying the *nos*ZII gene are more likely than those with *nos*ZI to also carry genes relating to dissimilatory nitrate reduction to ammonium (DNRA), a competing reduction pathway to denitrification [67]. Thus, the predicted abundance of *nos*ZI genes may be a better indicator of denitrification potential in oyster and sediment microbiomes than overall *nos*Z gene abundance.

Similar to other gene-based metabolic inference analyses, limitations exist regarding the quality and scope of the reference database being used as well as the understanding of the gene and metabolic pathways themselves. Our reference database was constructed with 5,445 complete and 222 draft bacterial genomes and curated for denitrification genes using KEGG or draft genome annotations. While the combination of these genomes covers a wide taxonomic range of bacteria, a great number of bacteria in many environments still remain unclassified or have identified genomes that are either incomplete or of low quality. Furthermore, caution must be used in inferring metabolic processes from gene presence in a bacterial genome. Often metabolic processes are extremely complex and require the coordinated expression of several different genes. While results from our study indicated that the relative abundance of the *nos*ZI gene is linked to denitrification potential of the oyster microbiomes, our study was small in scale and from only one season and location. Additional studies combining 16S rRNA gene studies and metabolic data are necessary to further validate the use of gene-based metabolic inferences as a reliable method for assessing the metabolic potential of microbiomes.

## Conclusions

By using a customized genome and denitrification gene database with the paprica program and 16S NGS data, we were able to characterize oyster microbiome structures and infer potential denitrifiers in the oyster digestive gland, shell, and sediment microbiomes. Phylotype comparisons of paprica with other taxonomic databases resulted in similar classifications of oyster microbiomes, providing reasonable confidence in gene inferences determined by paprica’s phylogenetic placement approach. Furthermore, qPCR of *nos*ZI genes were significantly and positively correlated with the *nos*ZI abundances inferred by paprica, providing additional evidence of reliability for gene inference. Overall, comparison of N_2_ fluxes with inferred denitrification genes from oyster digestive gland, shell, and sediment microbiomes suggest that increased denitrification activity in oyster reefs is driven by the increase of *nos*ZI gene-carrying bacteria, which may be important denitrifiers responsible for nitrogen removal in oyster reefs. Finally, this is the first study combining qPCR and N_2_ flux measurements to validate the use of 16S rRNA gene based metabolic inference as an alternative to whole genome sequencing in an effort to assess microbiome structure and connect microbiome function to the environment.

## Supporting information

S1 Fig Flowchart of bioinformatic pipeline. (TIF)

S2 Fig Relative abundances of bacterial families in shell (live) and shell (only) treatments. (TIF)

S3 Fig Relative abundances of *nos*ZI and *nos*ZII by taxonomical class. (TIF)

S1 Table Complete and draft bacterial genomes. (CSV)

S2 Table Customized gene database user file. (CSV)
