# Supplementary figures and images for: Denitrification potential of the eastern oyster microbiome using a 16S rRNA gene based metabolic inference approach
Source: PLoS One. 2017 Sep 21;12(9):e0185071. doi: 10.1371/journal.pone.0185071 (PMC5608302; doi:10.1371/journal.pone.0185071)

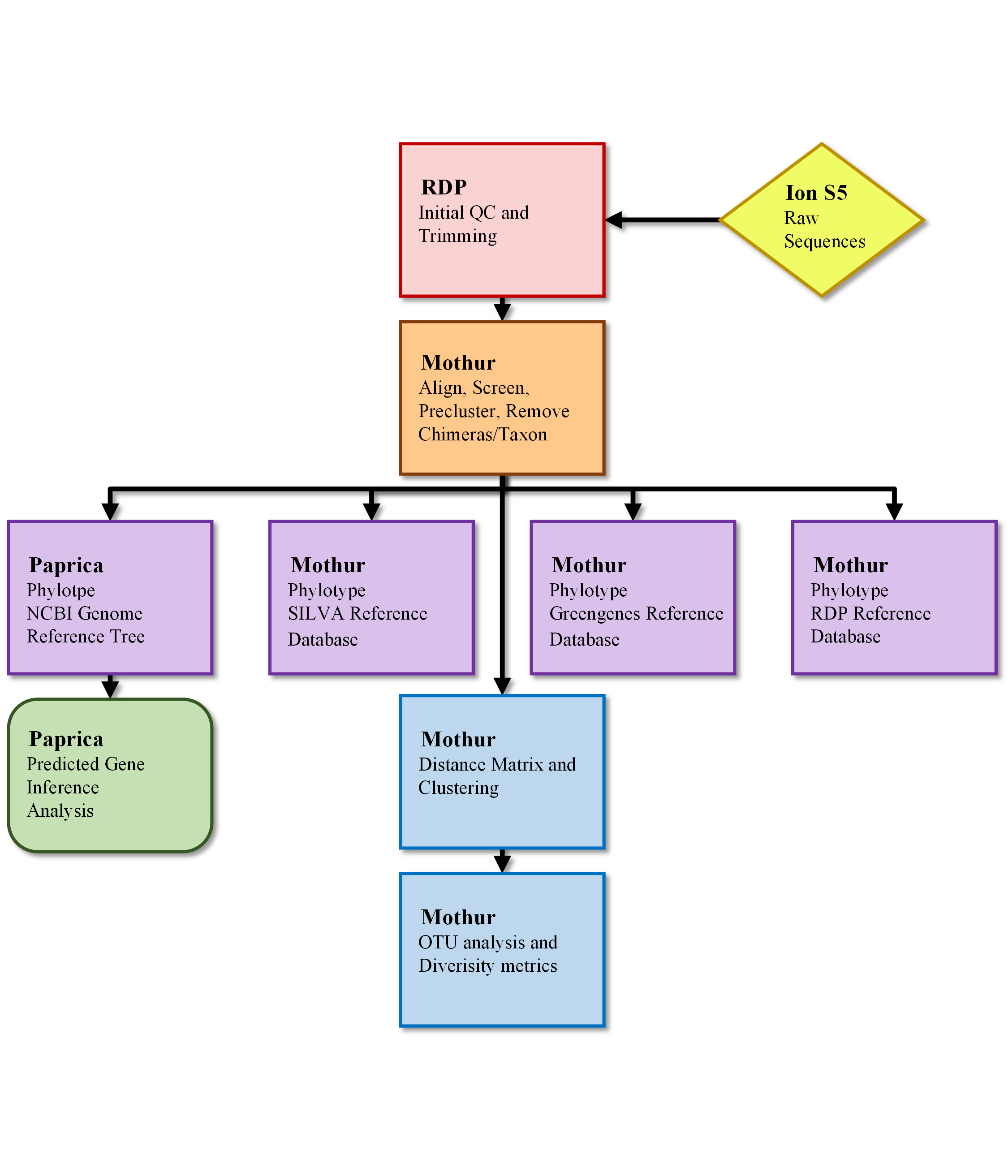

Supplement: S1 Fig — (TIF) [file pone.0185071.s001.tif]

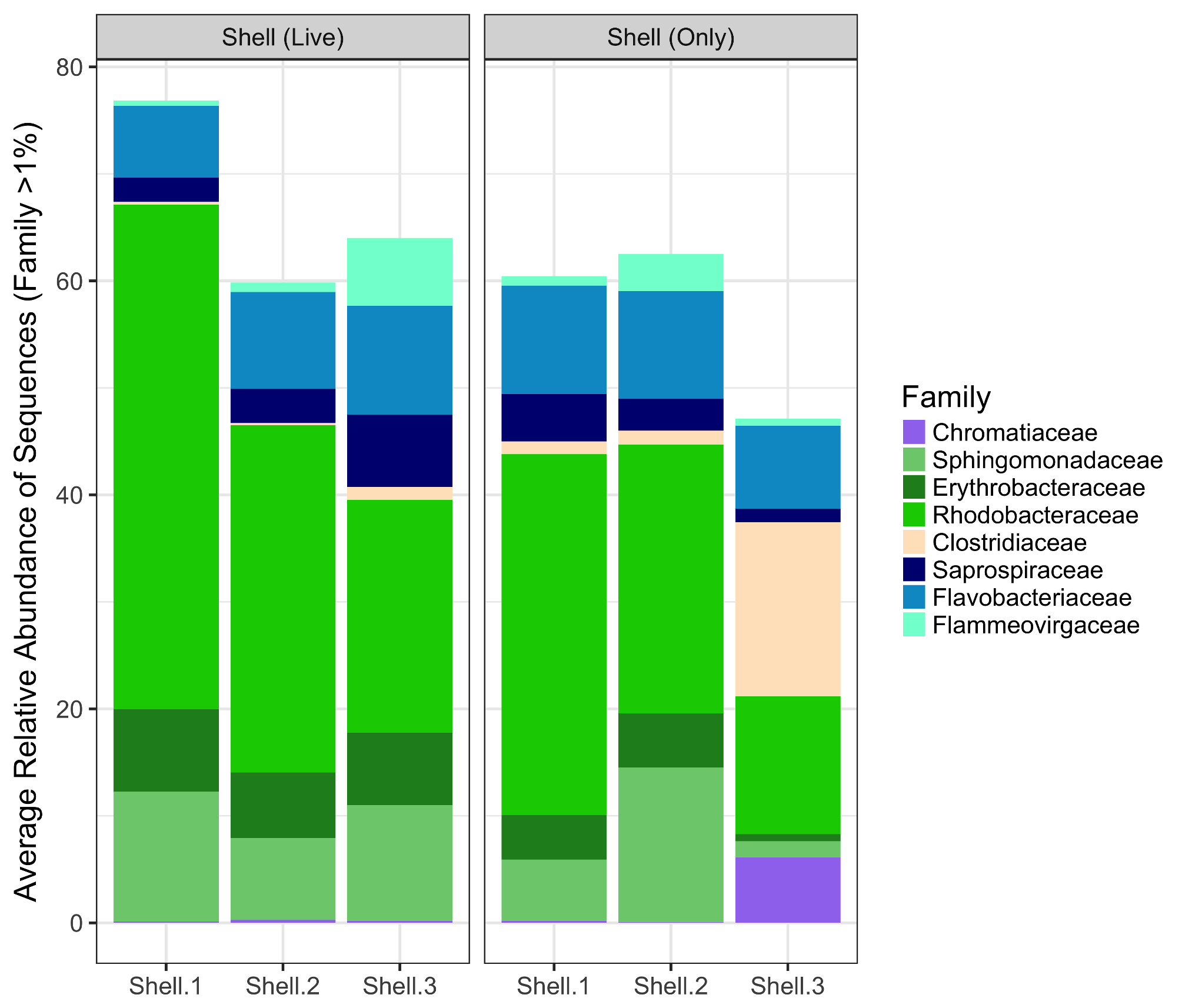

Supplement: S2 Fig — (TIF) [file pone.0185071.s002.tif]

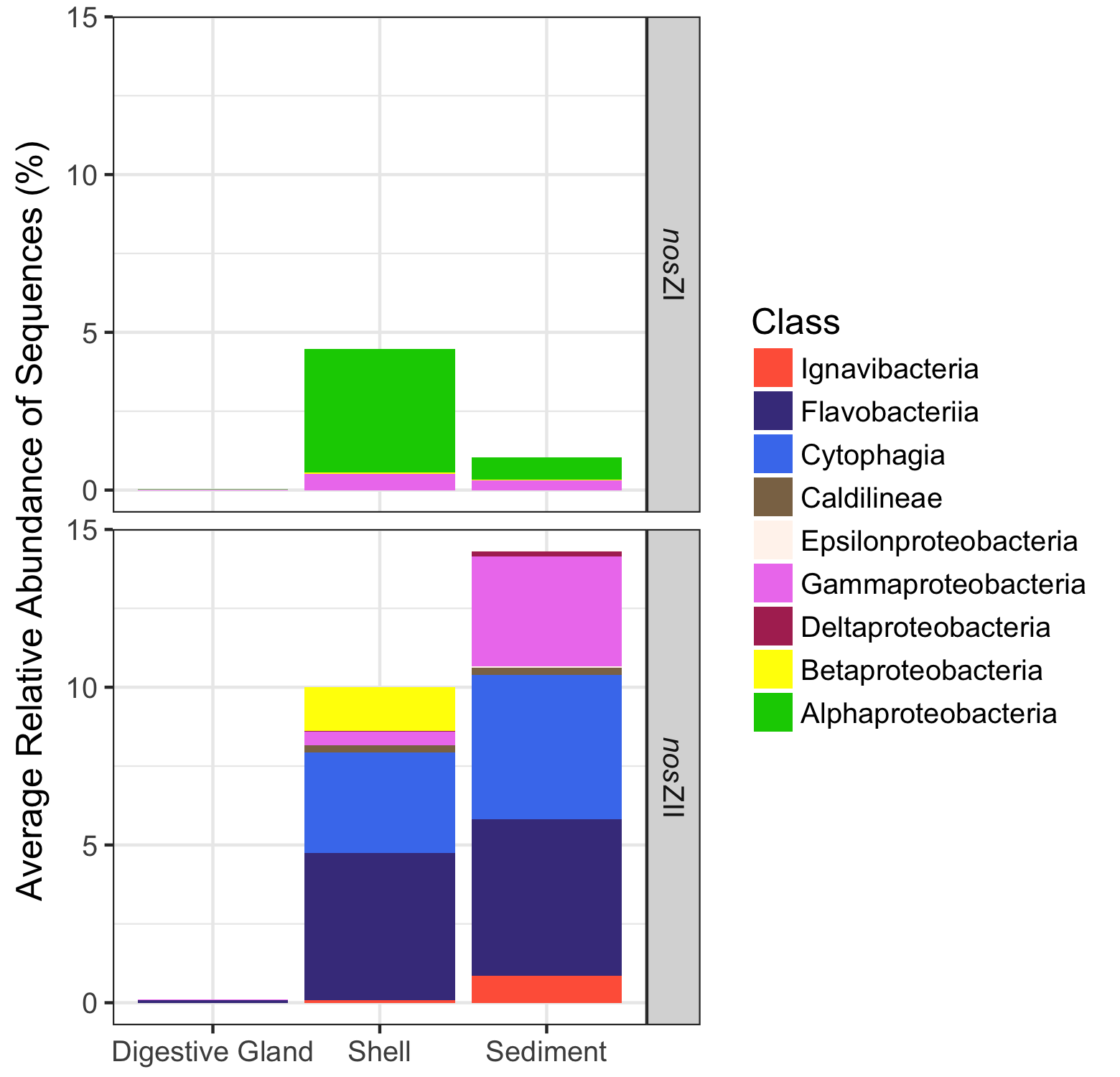

Supplement: S3 Fig — (TIF) [file pone.0185071.s003.tif]
